# Load-Bearing Assessment of Threads in 3D-Printed Polymer Elements

**DOI:** 10.3390/polym18010112

**Published:** 2025-12-30

**Authors:** Mateusz Śliwka, Błażej Wójcik

**Affiliations:** Department of Mechanical Engineering Fundamentals, Faculty of Mechanical Engineering and Computer Science, University of Bielsko-Biala, Willowa 2, 43-309 Bielsko-Biała, Poland; wojcik.blazej.wb@gmail.com

**Keywords:** threaded joint, 3D printing, SLS, PA12, experimental investigation

## Abstract

The article presents a comparative analysis of mechanical properties of M8 threaded joints produced using three different methods, in rectangular nylon (PA 12) specimens manufactured in SLS technology. Threaded holes in specimens were made by direct thread printing (specimens marked PT), thread reinforcement with Helicoil inserts (HT), and the use of heat-set inserts (IT). The specimens were subjected to a tensile testing at a constant displacement rate of 2 mm/min. The maximum force and the displacement at failure were recorded. The results indicated that the lowest load-bearing capacity *F*_MF_ was observed in the printed thread specimens, with an average value of 3.41 kN. The use of heat-set inserts increased *F*_MF_ to 3.83 kN, representing a 12% improvement. The highest load-bearing capacity was achieved in specimens reinforced with Helicoil inserts, which enhanced joint strength by 40% compared to printed thread specimens, reaching an average *F*_MF_ of 4.78 kN. In all cases, failure occurred due to the thread or insert pull-out from the specimen material. Studies have shown that the use of metal inserts significantly enhances the strength of threaded joints in SLS-printed PA12 components. Helicoil inserts provide the highest *F_MF_* load capacity, while heat-set inserts offer better technological advantages. Although printed threads are easier to manufacture, their applicability is limited to larger thread sizes and lower mechanical loads.

## 1. Introduction

3D printing technology is based on additive manufacturing, in which products are made by depositing material layer by layer [1,2]. Its key advantages include the reduced weight of printed components and the ability to produce parts with highly complex geometries [3]. The first commercial application of 3D printing dates back to 1980, when Charles Hull introduced the technology to the market [4]. Initially developed for rapid prototyping, it gradually transitioned to industrial-scale use as precision and technological capabilities improved [5]. Over the past decade, the field has expanded rapidly, largely due to the expiration of the earlier patent [2].

The use of 3D printing makes it possible to manufacture components directly from a previously created computer model (CAD) [6,7]. A wide range of materials can be used in the printing process, including plastics, metal powders, rubber, wood, sand, carbon fibres, and various organic materials [6]. In additive manufacturing, commonly used materials include thermoplastics such as polyamides (PA), polyimides (PI), polyetherimides (PEI), and polycarbonates (PC) [8]. Over the years, numerous 3D printing technologies have been developed, the main groups of which are presented below [9,10]:

1. Material Extrusion–layer by layer deposition of molten material (e.g., Fused Deposition Modelling–FDM, Polyjet).
2. VAT Photopolymerization–Selective hardening of photocurable material in a liquid container (e.g., Stereolithography-SLA, Digital Light Processing-DLP).
3. Material Jetting–material depositioning and subsequent curing (e.g., Multijet Printing-MJP).
4. Binder Jetting–Selective dispense of binder for joining powder in a bed (e.g., Sand Binder Jetting-SBJ, Multi Jet Fusion-MJF, Metal Binder Jetting-MBJ).
5. Powder Bed Fusion–Fusing of powder in a bed by melting the selected region (e.g., Selective Laser Sintering-SLS, Direct Metal Laser Sintering -DMLS).
6. Direct Energy Deposition–Direct fusion of the material (e.g., Wire Arc Additive Manufacturing-WAAM).
7. Sheet lamination–Bonding of individual sheets of material (e.g., Viscous Lithography Manufacturing-VLM).

Among additive manufacturing technologies, the most frequently and widely used are FDM and SLS [11]. SLS technology was developed in 1987 by Carl Deckard [10,12]. In SLS, a thin layer of powder is first spread across the platform using a scraper, after which the material is sintered by a laser beam [13]. The process is repeated—depositing successive layers of fresh powder and sintering them with the laser—until the entire object is formed [14].

Currently, 3D printing is applied in a wide range of industries, including aerospace [15], aviation [16], automotive [17,18,19], electrical and electronics [20,21,22], food [23], clothing [24], medical [25,26,27,28,29], defence [30], and construction [31].

In [32], the authors conducted experimental studies and FEM simulations on SLS-printed PA2200 polyamide paddle specimens, demonstrating that the print orientation significantly affects the material behaviour under load. Paper [8] examined 3D-printed polymer screws subjected to complex loads—tension, shearing, and their combination—considering the effects of print orientation and strain rate. The specimens were tested under quasi-static conditions, showing similar strength regardless of the print direction. In [1], the influence of the method used to make threaded holes in specimens on the load-bearing capacity of threaded joints was investigated. The authors of publication [33] conducted load-bearing tests on samples using fused metal inserts. The authors concluded that the print wall thickness should be at least 2.4 mm and the internal structure should be at least 70% filled. These printing parameters ensure high load-bearing capacity of the fused insert. In publication [34], static and cyclic compression tests were conducted on metric screws printed using FDM technology. Based on the results, the authors concluded that despite uniform production conditions for threaded elements using the FDM method, it is difficult to clearly predict the nature of damage or the value of the breaking stress. Therefore, it is advisable to consider the safety factor when using polymer bolted connections. In [35], experimental studies of the self-loosening of M12 threaded connections were conducted on three different materials under the influence of cyclic temperature changes.

Threaded holes have broad applications, including in household appliances and electronics, such as housings for mixers and television sets. In the automotive industry, they are used in dashboards, lamp housings, and air filter enclosures. Additionally, they find applications in furniture, lighting, and toy manufacturing.

## 2. Materials and Methods

The study used printed cuboidal specimens measuring 35 × 35 × 25 mm, each containing an M8 threaded hole made using three different methods. The specimens were printed from PA12 nylon using SLS technology on a Formlabs Fuse 1 printer [36]. The layer thickness was 0.11 mm.

### 2.1. Specimens with Printed Thread (PT)

**Figure 1:**
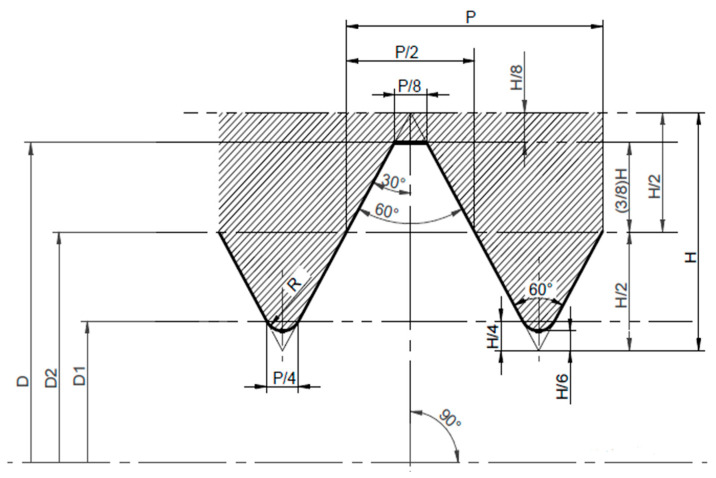
Shape and dimensions of the thread [1].

In the first type of specimens, the M8 thread was printed. These specimens are designated PT. The 3D model of the printed thread specimen was prepared based on the metric thread profile shown in Figure 1.

The dimensions and shape of the threaded hole shown in Figure 1 are summarized in Table 1.

### 2.2. Specimen with Helicoil Thread (HT)

**Figure 2:**
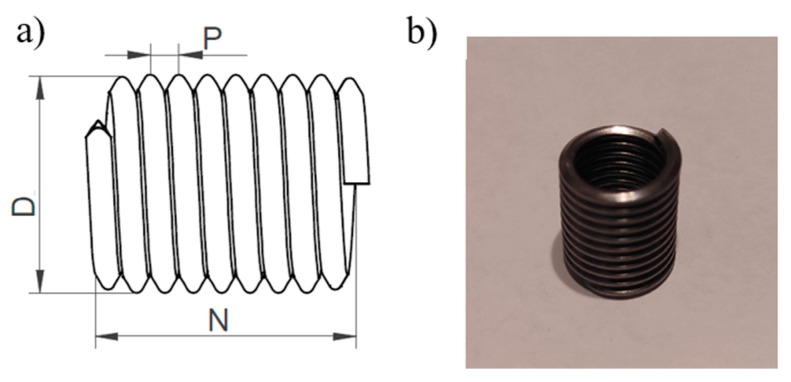
Helicoil insert (**a**) insert drawing, (**b**) insert before assembly.

The next type of specimens had threads reinforced with a Helicoil insert. It is a component used in mechanical engineering to repair or strengthen threads in damaged or weak materials. This type of inserts was developed to address problems associated with thread damage caused by failure, abrasion, or wear resulting from repeated assembly and disassembly. They are spirally wound inserts made of high-quality materials, such as stainless steel, and are designed to provide durable and reliable threaded connections in a variety of applications. Figure 2a shows a drawing of a Helicoil insert, while Figure 2b presents an M8 Helicoil insert.

**Figure 3:**
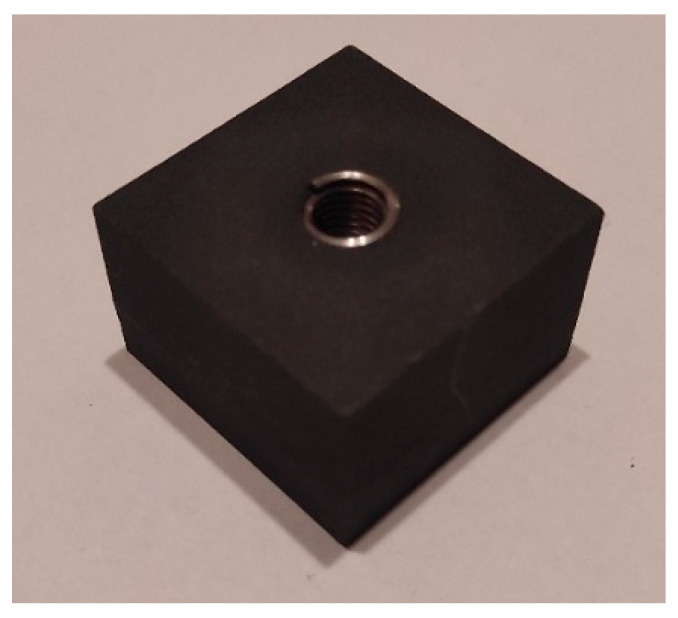
Specimen with Helicoil insert.

Reinforcing the M8 thread with a Helicoil insert required printing M9 × 1.25 threaded hole in the specimen. The M8 Helicoil inserts were then screwed into these holes. Figure 3 shows the specimen after the insert had been installed.

### 2.3. Specimen with Insert Thread (IT)

**Figure 4:**
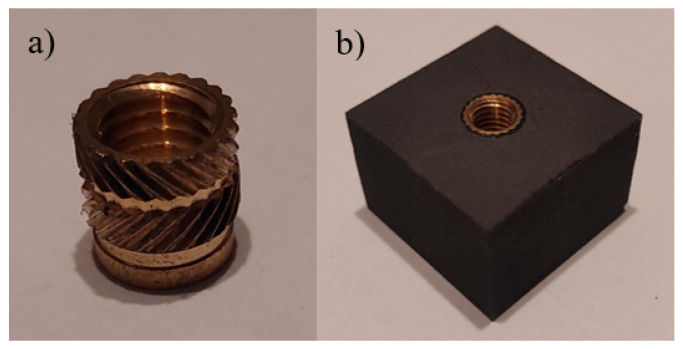
(**a**) M8 Insert, (**b**) specimen with heat-set insert.

The third type of specimens was prepared using a solution designed for threading polymer parts produced by 3D printing. This specimen type was designated IT. Threaded inserts were installed in the prepared holes by embedding them into the print using a soldering iron or suitable tooling from various manufacturers. Even a low temperature is sufficient to permanently secure the insert in the component; however, the embedding temperature should be adjusted according to the material of the specimen. For an M8 threaded insert (Figure 4a), a hole with a diameter of 9 mm should be prepared for embedding the insert. The resulting specimen is shown in Figure 4b.

### 2.4. Experimental Studies

**Figure 5:**
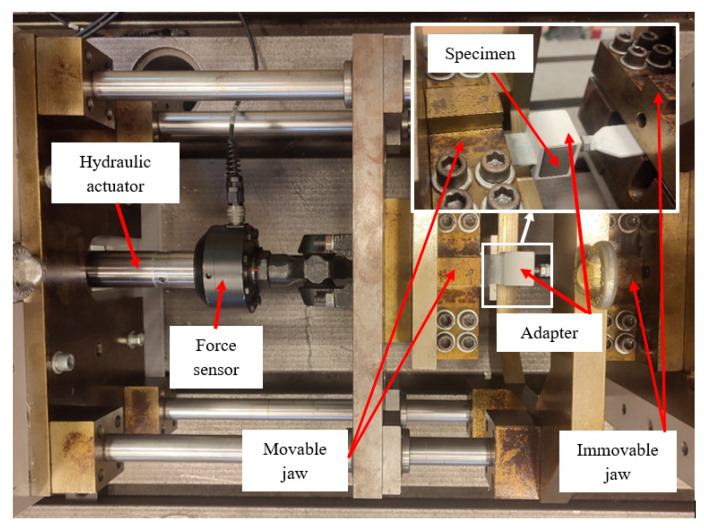
Testing machine with a mounted specimen.

Thread load-bearing capacity tests were conducted using an MTS measuring system, which included a hydraulic actuator with a ±75 mm working range and a ±25 kN force capacity, a force and displacement sensor and appropriate mounting adapters. The machine’s accuracy class is 0.5. Each specimen was secured in a mounting adapter which was placed in the immovable jaw of the machine. A class 3.6 screw was inserted into the threaded hole to a depth of 8 mm, and the other end of the screw was attached to the movable jaw. The testing machine with the mounted specimen is shown in Figure 5.

For each of the three types of connections, a thread load-bearing capacity test was performed in a tensile test at a speed of 2 mm/min. Five tests were performed for each thread type, with the maximum force and corresponding displacement at failure recorded. The selection of 5 specimen was due to high costs, and the experiment was conducted on a measuring machine with high measurement accuracy.

## 3. Results and Discussion

During the tests, the force was recorded as a function of the actuator displacement of the measuring system.

### 3.1. PT Specimens

**Figure 6:**
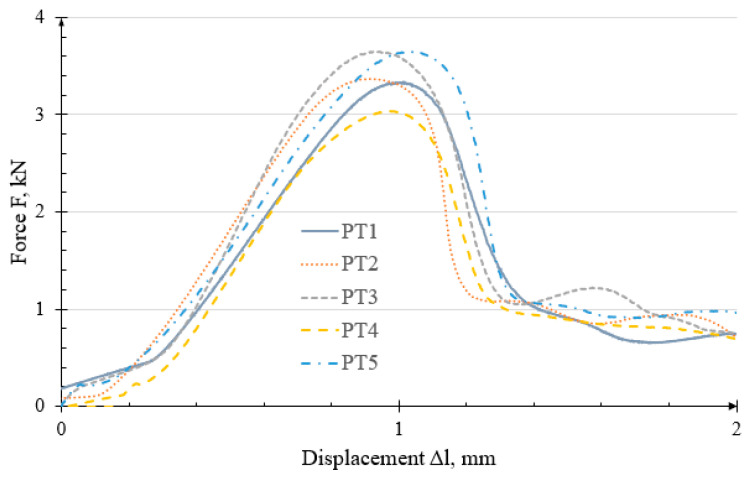
Characteristics of the failure curves of the PT threaded joint.

Load-bearing capacity tests were conducted on specimens with printed threads. The results, presented as force–displacement characteristics F(Δl), are shown in Figure 6.

The characteristics recorded during testing varied between specimens. This variability may be attributed to limited repeatability and accuracy during printing, as well as possible differences in thread dimensions or profiles, which affected the measured failure forces. The maximum force $F_{\mathrm{MF}}\;$ recorded during the test was taken as the failure criterion. The average failure force was 3.409 ± 0.320 kN, and the joint failed at an average displacement of 0.977 ± 0.091 mm. The trend for all specimens is similar. The force increases bilinearly, then reaches an extreme and then drop, stabilizing at approximately 1 kN.

Failure of the threaded joint occurred due to the threaded hole being pulled out of the printed part. No other damage was observed on the specimen surfaces, apart from the torn thread coils.

### 3.2. HT Specimens

**Figure 7:**
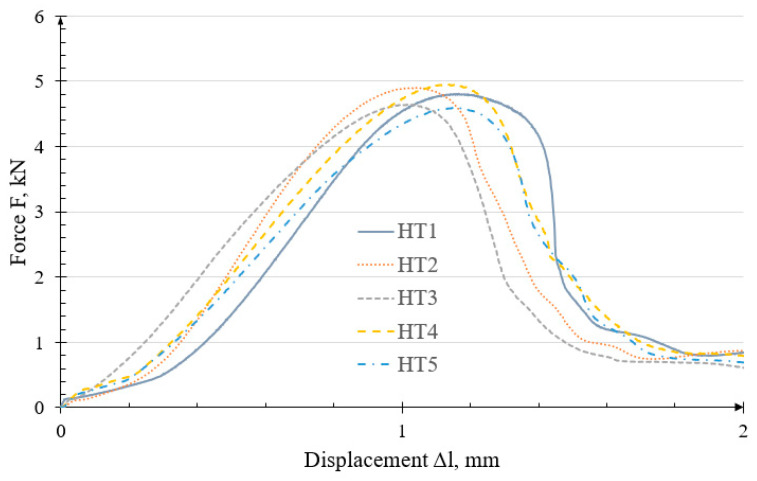
Characteristics of the failure curves of the HT threaded joint.

Testing was continued on specimens reinforced with Helicoil inserts. Figure 7 presents the load-bearing capacity graph for the HT specimens. The force curves for the HT specimens are similar to the PT trends.

For the HT specimens, the failure mechanism was consistent, as indicated by the similar characteristics. This suggests that any inaccuracies or internal defects arising during the 3D printing process did not significantly affect joint strength. The average breaking force was 4.779 ± 0.288 kN. An additional important observation is that the use of a Helicoil insert produced a joint with greater resistance to abrasion and wear during repeated screw tightening and loosening. The joint failed at an average displacement of 1.103 ± 0.095 mm.

**Figure 8:**
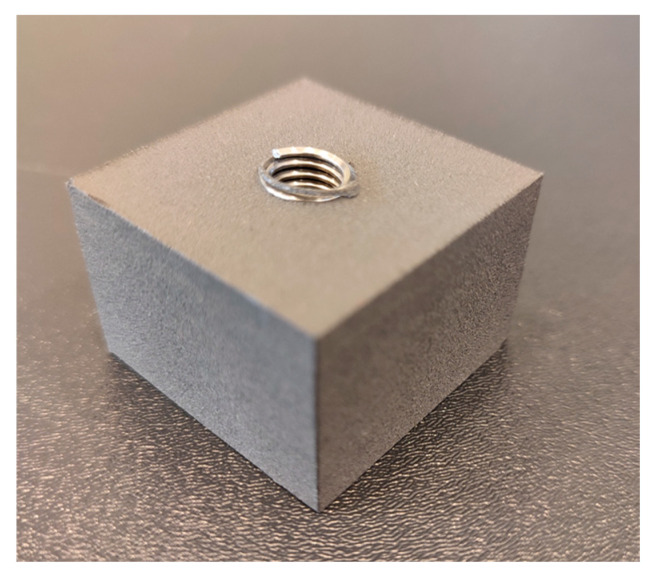
Specimen after testing.

The failure occurred due to the Helicoil insert being pulled out of the hole (Figure 8).

No other damage was observed on the entire surface of the specimens, except for the fact that thread coils, where the insert was placed, were pulled out.

### 3.3. IT Specimens

**Figure 9:**
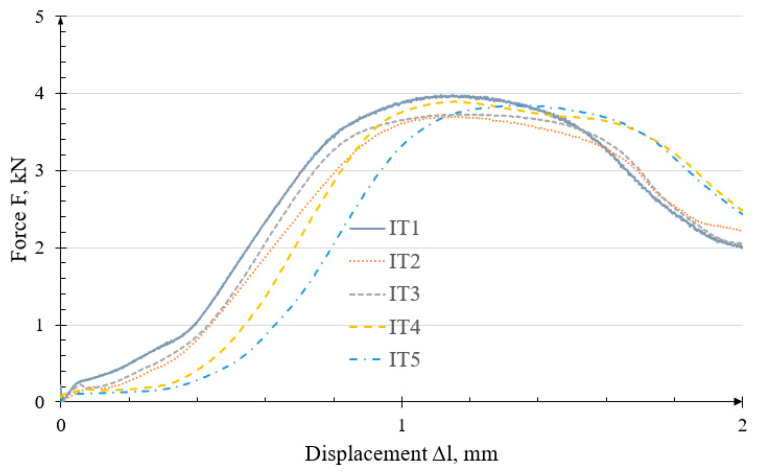
Characteristics of the failure curves of the IT threaded joint.

Further testing was conducted on the IT specimens. The corresponding test graph is presented in Figure 9.

The characteristics are comparable, with an average force of 3.827 ± 0.150 kN. The force increase is nonlinear, and after reaching the maximum value, the force decreases gently, reaching approximately 2 kN at a displacement of 2 mm. This result is significantly worse as compared to the other solutions. The strength of this application depends primarily on the insert assembly process, which is performed manually. Variable conditions also arise during this procedure, which consequently leads to different expected outcomes. The maximum load-bearing force of the IT connection was observed at a displacement of 1.174 ± 0.215 mm.

The failure of the threaded joint occurred when the insert was pulled out of the printed specimen, with no other damage observed on the specimen surfaces.

The failure force $F_{\mathrm{MF}}$ and the corresponding displacement Δl  for all specimens are summarized in Table 2.

**Figure 10:**
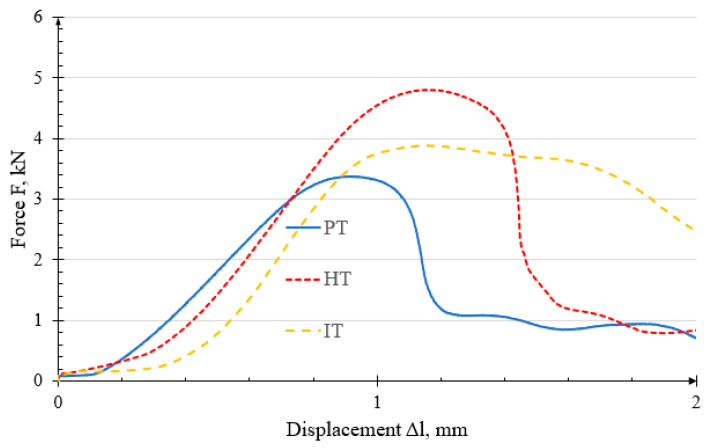
Comparison of force-displacement characteristics.

From the tests conducted on each specimen group, a single representative characteristic was selected for each group. Figure 10 presents a graph summarizing these representative characteristics.

The PT and HT curves exhibit similar characteristics, although the HT specimens show a 1.37 kN higher load-bearing capacity. The average failure force of the IT specimens is 0.952 kN lower than that of the HT specimens.

## 4. Conclusions

Analysis of the results shows that the lowest failure force occurred in the specimens with printed threads (PT). Incorporating metal inserts with a pre-formed internal thread increased joint strength by 12.3%, while the use of a Helicoil insert (HT) enhanced the load-bearing capacity by as much as 40.2%.

The HT and IT specimens failed at a displacement of approximately 1.1 mm. In contrast, the PT specimens reached their maximum force at a displacement approximately 0.2 mm lower than that of the other specimens.

The strength tests indicate that the use of dedicated plastic inserts improved joint performance by just over 10%. In these cases, the failure mode was limited to the insert being pulled out of the specimen. A key advantage of such inserts is their suitability for applications requiring small thread sizes, such as M2 or M3. However, a drawback of the heat-set insertion process is the tendency of material to bulge on the top surface of the insert. This defect must be manually removed, and the surface smoothed, to ensure proper joint preparation. The inserts are made of hard materials, which suggests high wear resistance of the threaded joint—a topic that will be addressed in future studies.

The use of Helicoil inserts requires an appropriately sized thread to be created in the hole beforehand, with the thread type and parameters specified by the insert manufacturer. If the thread is not made during the printing process, it must be cut manually using a suitable tap. However, this manual operation can damage the printed structure and consequently weaken the threaded joint.

It is important to note that printing threads is feasible only for sufficiently large thread dimensions, typically those greater than M6. Smaller threads cannot be reproduced with adequate accuracy, which may lead to a weakened joint.

## Figures and Tables

**Table 1:** M8 thread dimensions in the analysed group of specimens.

Name/Type	Symbol	Value	Unit
Height	H	1.0825	mm
Distance	H/2	0.5413	mm
	3/8H	0.4059	mm
	H/4	0.2706	mm
	H/6	0.1804	mm
	H/8	0.1353	mm
Nominal thread diameter	D	8.00	mm
Pitch	P	1.25	mm
Radius	R	0.18	mm
Thread angle	Alfa	60	
Major nominal diameter	D	8	mm
Flank diameter	D2	7.1888	mm
Core diameter	D1	6.6475	mm
Thread depth	H1	0.6766	mm
Profile bottom chamfer	P/8	0.1563	mm
Profile tip chamfer	P/4	0.3125	mm
Pitch half	P/2	0.6250	mm

**Table 2:** Results comparison.

Number	Load-Bearing Capacity *F_MF_*, kN			Displacement Δl, mm		
	PT	HT	IT	PT	HT	IT
Specimen 1	3.330	4.806	3.973	1.044	1.153	1.151
Specimen 2	3.373	4.906	3.697	0.916	1.041	1.041
Specimen 3	3.653	4.644	3.727	0.925	1.036	1.172
Specimen 4	3.036	4.949	3.895	0.959	1.137	1.161
Specimen 5	3.653	4.591	3.845	1.042	1.147	1.367
Average	3.409	4.779	3.827	0.977	1.103	1.174
Standard deviation	0.258	0.181	0.094	0.057	0.060	0.135
Kurtosis	−0.498	−5.307	−4.203	1.737	−5.827	1.839
Skewness	−0.569	−0.025	0.151	1.440	0.018	0.811
Min Force	3.036	4.591	3.697	0.916	1.036	1.041
Max Force	3.653	4.949	3.895	1.042	1.147	1.367
Confidence interval	0.320	0.288	0.150	0.091	0.095	0.215

## Data Availability

The raw data supporting the conclusions of this article will be made available by the authors on request.
